# Clinical Outcomes of Angiotensin Converting Enzyme Inhibitors and Angiotensin II Receptor Blockers in COVID-19 Patients With Pre-existing Cardiac Comorbidities: A Literature Review

**DOI:** 10.7759/cureus.51244

**Published:** 2023-12-28

**Authors:** Sandhya Srinivasa, Simran Kaur, Anam Dharani, Ellen Choi, Amar Kalidas, Robert Slater, Steven Mifflin

**Affiliations:** 1 Medicine, University of the Incarnate Word School of Osteopathic Medicine, San Antonio, USA; 2 Integrative Medicine, University of the Incarnate Word School of Osteopathic Medicine, San Antonio, USA; 3 Research and Development, University of the Incarnate Word School of Osteopathic Medicine, San Antonio, USA

**Keywords:** congestive heart failure, hypertension and covid-19, angiotensin ii receptor blockers (arb), angiotensin converting enzyme inhibitors (acei), covid 19, sars-cov2 infection

## Abstract

The growing research regarding the implementation of angiotensin-converting enzyme-2 inhibitors (ACEi) and angiotensin receptor blockers (ARBs) in the treatment of COVID-19 in patients with pre-existing cardiac comorbidities has become a large topic of discussion since the onset of the pandemic. Previous research primarily associates positive outcomes to the use of these drug classes due to their mechanism of action, which involves the downregulation of angiotensin I-converting enzyme 2 (ACE2) in the renin-angiotensin-aldosterone-system (RAAS) pathway, inflammatory mediators, and cytokines. Thus, these medications can convey preventative and protective effects in patients suffering from a SARS-CoV-2 infection. While we explored the studies that supported the positive outcomes of the use of these drugs in the first half of this review, we also expanded on the limitations of these studies in the latter portion. We also further explored the contradictory studies that indicated that using these antihypertensives can paradoxically increase the severity of COVID-19 infection as well. The studies in support of the use of these medications should consider epigenetic variations, ACE2 variants and acknowledge inherent genetic variations in certain ethnic groups as some have a predisposition for a severe COVID-19 infection. Additionally, mortality rates need to be taken into consideration in these studies as they naturally differ throughout the trajectory of the COVID-19 pandemic. While some studies are in support of the use of these antihypertensives despite other studies suggesting otherwise, further research is needed to explore the long-term effects of these antihypertensives and observe whether they are truly beneficial or not in reducing the severity of COVID-19 infections.

## Introduction and background

Physiology and regulation of ACE and RAAS activity

The renin-angiotensin-aldosterone system (RAAS) plays a role in regulating the volume of blood and vascular resistance. The RAAS system responds to changes in arterial pressure, decreased renal pressure, and decreased salt delivery to the distal convoluted tubule. In response to these changes, the juxtaglomerular cells are activated, which then cleaves prorenin to renin. Once released into the blood, renin will target and cleave angiotensinogen into angiotensin I. Angiotensin-converting enzyme (ACE), found primarily in the lungs and kidneys, will convert angiotensin I into angiotensin II by binding to angiotensin II type I and type II receptors. Angiotensin II binding at the proximal convoluted tubule of the renal tubule, through mechanisms of transport and reabsorption, will then result in increased osmolarity of the blood and, thus an increase in blood volume and arterial pressure. Angiotensin II at the adrenal cortex stimulates aldosterone, which will then increase the salt reabsorption to increase blood and extracellular fluid volume [1]. 

ACE is a major regulator in the cardiovascular system and is the target of several pharmaceuticals, namely angiotensin-converting enzyme-2 inhibitors (ACEis) and angiotensin receptor blockers (ARBs), in treating cardiac and respiratory conditions, such as congestive heart failure and hypertension. In addition to cleavage of the angiotensin I to II, ACE is also responsible for the breakdown of bradykinin, a vasodilator. With the changes and developments seen through the era of COVID-19, the importance of ACE2 in maintaining homeostasis, as well as its direct interaction with SARS-CoV-2 becomes more prominent. Recent reviews highlight the role of ACEis and ARBs in upregulating ACE2 expression and the potential consequential effects for COVID-19 patients [1]. 

Effects of ACE2, ACEis, and ARBs within the pulmonary system

SARS-CoV-2 leads to the downregulation of ACE2 and the angiotensinogen, ang-(1-7), molecules within endothelial and lung type 2 alveolar epithelial cells [2]. Given these effects, it has been proposed that ACEis and ARBs can be beneficial by blunting the downregulation of ACE2 expression. In animal models infected with SARS-CoV-2, ACE2 downregulation resulted in increased inflammatory responses, leading to an increase in lung injury and impaired cardiac contractility. Decreased levels of ACE2 lead to a reduction of ang-(1-7), and this reduces vasodilatory and anti-inflammatory effects by decreasing the release of interleukin (IL)-6 and tumor necrosis factor (TNF)-α, reducing macrophage infiltration, and increasing the release of nitric oxide [2].

Although ACEis and ARBs do not directly affect the expression of ACE2, these drugs can indirectly increase the activity of ACE2 and ang-(1-7) expression. Ferrario and co-authors, in their paper, show that ACEis lead to significant anti-inflammatory effects by increasing ang-(1-7) concentrations [3]. 

Given previous research, the usage of ACEis and ARBs may have potential benefit in preventing COVID-19-triggered organ damage, given the drug's ability to upregulate ang-(1-7) and deplete angiopoietin-2 (ang-2). Decreased ACE2 has been shown to worsen the decline in lung function and has injurious cardiovascular effects. Thus, despite acting as a receptor for SARS-CoV-2, ACE2 activity is likely protective in preventing severe disease progression and clinical deterioration following COVID-19 infection, reflecting the drug's effect on abrogating the inflammatory response and vasoconstriction [4].

Effects of ACEi in patients with cardiac system comorbidities

ACEis and ARBs have been used in cardiac patient populations due to their beneficial effects in managing cardiovascular disease. The benefits include improving hemodynamics, reducing congestive heart failure symptoms, reducing diuretic requirement, and decreasing mortality and hospitalization. ACEis have acute and sustained hemodynamic effects in patients with left ventricular dysfunction. They act by increasing cardiac output and stroke volume while reducing systemic vascular resistance. This will consequently improve any signs and symptoms that are related to congestive heart failure. They exert these effects through regulating the RAAS pathway [5]. 

A review article by Khalil et al. cited clinical trials demonstrating that the treatment with ACEis in patients with left ventricular dysfunction antagonized the neurohormonal activation, which slowed down the progression to a pump failure. Acute myocardial infarction or cardiac muscle damage can be seen in nonischemic cardiomyopathy, and the compensatory mechanisms are activated to maintain cardiac output. Some of the compensatory mechanisms include maintaining a sympathetic tone and activation of RAAS. Sympathetic tone will help to increase the contractility of the remaining heart muscles. It will also increase systolic and diastolic wall stress, which will stimulate myocytes to hypertrophy and help return the wall stress back to normal. ACEis will help these patients by improving myocyte contractility and preventing non-myocyte cellular proliferation and collagen deposition [6].

Elevated renin-angiotensin aldosterone levels can promote fibrosis and collagen deposition and lead to progressive left ventricular dysfunction. ACEis can be beneficial in this case by increasing plasminogen activator inhibitor-1 (PAI-1) to inhibit endogenous fibrinolysis. With respect to preventing ischemic events, ACEis plays a role in counteracting vasoconstriction of the atherosclerotic coronary arteries and preventing endothelial dysfunction. It does so by counteracting the processes that involve thrombosis, low-density lipoprotein oxidation, and local accumulation of neutrophils. ACEis release nitric oxide and bradykinins in addition to counteracting the effects of angiotensin II by inhibiting angiotensin-converting enzymes [6].

Methods

We have compiled a total of 26 articles during the process of writing this literature review. The articles describe the physiological explanation of ACE2 expression and its relation to COVID-19 and cardiomyopathies and specific drugs that could potentially have beneficial effects on COVID-19 patients. We also researched different populations that may be affected by the use of these medications. Articles that provided contradictory evidence and exposed the limitations of the studies were also explored. 

## Review

Effects of ACEi in cardiac patients with COVID-19

SARS-CoV-2 infection has caused a pandemic with serious outcomes, major morbidity, and increased mortality. This virus attaches to ACE2, which is found extensively in alveolar tissue and myocardial tissue. ACE2 is a cardioprotective transmembrane protein whose expression is downregulated by SARS-CoV-2 infection [6]. Overall, it has been concluded from numerous research articles that ACEis and ARBs have a more protective than harmful effect in terms of COVID-19 outcomes. In COVID-19 infection, the cardiac expression of ACE2, the target for the SARS-CoV-2 spike protein, is implicated in the pathophysiology of the associated myocardial injury [6]. Specifically, SARS-CoV, from the 2003 outbreak, and SARS-CoV-2 have a similar spike protein virulence factor, which allows for entry into a cell via recognition of the ACE2 receptor. SARS-CoV-2 binds the ACE2 receptor more efficiently than SARS-CoV. This explains an increase in virulence and transmission during this pandemic. Furthermore, varying levels of ACE2 expression have been found in different populations, which could contribute to the more damaging cardiac effects seen in some patients, such as the African population [7]. 

Physiologically, ACEis and ARBs raise the ACE2 mRNA expression. ARBs, namely olmesartan, losartan, valsartan, candesartan, telmisartan, and irbesartan, can all increase ACE2 protein by about two-fold in the hearts of aorta-constricted mice. Similar to ARBs, ACEis, such as enalapril and lisinopril, also raise ACE2 mRNA expression and/or protein levels or activity in the plasma, heart, and kidney of animals [8]. This finding argues that certain commonly prescribed drugs may affect the RAAS pathway by increasing ACE2 expression. Other drugs, such as the diabetic drug class glucagon-like peptide 1 (GLP-1), may also have similar effects on ACE2 expression, meaning a wider population could benefit from these commonly prescribed drugs. Dalbavancin, a GLP-1, is shown to inhibit SARS-CoV-2 spike protein-ACE2 interactions by directly binding to ACE2 [9]. This shows that other drugs besides ACEi and ARBs, through a shared mechanism, can contribute to the cardioprotective effects. Opting to increase prescribing of certain drugs for common conditions can benefit overall cardiac health, especially since heart disease is a common condition. Therefore, it may be beneficial to move towards using these drugs in the aging population.

Determining cardioprotective drugs and their mechanisms of action are important because the advanced phases of COVID-19 are characterized by a cytokine storm whose profile seems to be similar to the one encountered in other viral infections. The cytokine storm causes an increase in IL-3, IL-6, IL-7, granulocyte-colony stimulating factor, interferon-γ inducible protein 10, monocyte chemoattractant protein 1, macrophage inflammatory protein 1-α, and TNF-α and ferritin. These characteristics can stress the heart via cytokine-related myocardial dysfunction [10]. ACEi and ARB use may prevent this cytokine storm from occurring by decreasing these inflammatory mediators. Additionally, the cytokines mentioned above that arise due to the condition increase blood viscosity and coagulability. This causes endothelial dysfunction and promotes electrolyte and hemodynamic imbalance [11]. It appears that a pre-existing cardiac condition and the new onset of COVID-19 create a myriad of symptoms that lead to an eventual downfall. The same article claims that when pulmonary complication and hemodynamic instability prevail, COVID-19 is associated with diastolic dysfunction, specifically while systolic dysfunction subsequently increases as a consequence of the cytokine effect. This speaks to more specific cardiac dysfunctions that may prevail after COVID-19 [11]. 

The same article above describes a study of 17 patients treated in Shenzhen, Third People's Hospital. They were treated with multiple different drugs, including ACEis and ARBs. The study found the ACEi and ARB group to have less severe disease and lower levels of IL-6, a higher absolute number of CD3+ and CD8+ T cells, and a lower peak viral load during hospitalization. Results of the study show that specifically losartan has shown a protective role [11]. Pinpointing specific ACEis and ARBs is a monumental step in determining which ones may have additional benefits over others. This can streamline the process of prescribing medications for certain patients with cardiac comorbidities susceptible to COVID-19. 

Another study explored the effects of ACEis on reducing COVID-19 morbidity in patients with hypertension with data organized based on insurance coverage of Medicare Advantage and commercial insurance. In the study, it was reported that of the 2263 COVID-19 patients who had associated hypertension, those who received treatment with ACEis were 40% less likely to be hospitalized than if they were not treated with the medication [12]. This shows the positive effect of these ACEis on preserving cardiac health while also controlling for other comorbidities, particularly hypertension, and how it can provide additional benefits in slowing the progression of COVID-19. While this initial study showed the potential benefits of ACEi use, a followup study of an inpatient cohort of 7933 patients suggested that there is no correlation between ACEi use and COVID-19 hospitalization rates. Additionally, a lower risk of hospitalization was seen in the Medicare group, but this was not seen in the younger patient population with commercial insurance. Given the recent findings showing inconsistencies with previously reported data, the role of ACEi and ARBs still remains arguable [13].

Counterargument and further studies

While the previous half of this literature review discussed studies that supported the utilization of ACEis and ARBs in patients with cardiac comorbidities as a mechanism of protection against COVID-19, we will now discuss the potential limitations of these studies and research that show that ACEi and ARB use can also potentially convey a worse prognosis for patients with COVID-19 infection. We will first discuss genetic variations in various ethnic groups and epigenetic variations and how they present as a limitation. Then, we will explore the debate on the mechanism of ACEis and ARBs on ACE2. Lastly, we will discuss why variations in mortality at different points in the trajectory of the pandemic are a potential limitation. 

The varying effects of ACEis and ARBs on COVID-19 in different ethnicities could present as a confounding variable when studying the benefits of these antihypertensives in reducing COVID-19 severity. Based on population-wide results from these studies, it is possible that certain populations have a genetic predisposition for contracting severe COVID-19 infections regardless of whether they are taking ACEis and ARBs. In one study, researchers analyzed pulmonary cells through single-cell RNA sequencing and found higher ACE2 levels in the lungs of Asians compared to Caucasians and Africans [14]. Another study found that Saudi Arabians had a higher risk of COVID-19 from ACEi and ARB use [15]. Others found that African Americans taking these medications were less likely to experience COVID-19-related mortality in comparison to other non-African populations [16]. However, another study in the Italian population stated that COVID-19 severity was unchanged with ACEi and ARB intake. This population-wide variation in the effects of these medications seen in these studies can be attributed to differences in socioeconomic status, lifestyle, and cultural differences. However, it could also be due to variabilities in ACE2 gene alleles in these populations or ACE2 gene expression. Studies have also found that the African American population has a lower ACE2 gene expression in comparison to the Caucasian population [17]. Studies must take into consideration the race of the patient when examining COVID-19 severity; this could be a potential limitation to the previously mentioned studies that examined the benefits of ACEi and ARB use in reducing COVID-19 disease severity. 

While the previously mentioned studies focused on population-wide variation in ACE2 expression affecting COVID-19 disease severity, there could also be individual differences in COVID-19 severity as well. Studies have shown that, within the Italian population, there are many different ACE2 variants [18]. Therefore, even within the population, some individuals have a greater genetic predisposition for a severe COVID-19 infection compared to others. This could be a characteristic of populations other than simply the Italian population. Future research should focus on determining the prevalent ACE2 variants in each population. This individualized difference is also not taken into account in the studies mentioned in the first half of this review. 

In addition to variations in ACE2 expression and ACE2 variants, there are also other epigenetic factors at play when determining which individuals have a predisposition for severe COVID-19 infection. When studying the African American population, researchers noted that they have a decreased ACE2 expression but still experienced higher than normal rates of COVID-19 infection; this is contraindicatory to the expected decreased risk of COVID-19 in this group due to lower ACE2 expression. Researchers attribute these unexpected results to epigenetics processes like methylation and single nucleotide polymorphisms [8]. It is essential that the previously mentioned studies that supported the use of ACEis and ARBs in COVID-19 patients with cardiac comorbidities take into consideration epigenetics, ACE2 variants, and differences in ACE2 expression in ethnic groups. This is a potential limitation in the previously mentioned articles and is an area that requires further research. 

Despite the presumed benefit of using ACEi and ARB therapy for COVID-19 patients that was discussed earlier in this review article, there have been some contradictory studies that have shown that these drugs could also potentially worsen COVID-19 infection. Some studies suggest that ACEi use can paradoxically upregulate ACE2 expression, therefore increasing the quantity of ACE2 receptors, which can facilitate more host cell entry [19]. This is contradictory to the studies showing that ACEi and ARB use can provide cardioprotective effects with exposure to COVID-19. However, it is important to understand that even though there have been retrospective observational studies, such as Guo et al.'s, which showed an association between using ACEis and worsened outcome of COVID-19, it is difficult to confirm causation as ACEi use can be a confounding factor that can indicate comorbidities [20]. Thus, the overall long-term benefits and use of ACEis and ARBs for reducing COVID-19 severity are still a topic of debate. 

One particular study discusses how the use of ACEis can potentially be both beneficial and detrimental depending on the phase of COVID-19 infection. During earlier stages, increased ACE2 expression can lead to increased entry of COVID-19 into host cells, and this increase in ACE2 expression can be seen in patients taking ACEis and ARBs. In later stages, COVID-19 infection can cause a decreased ACE2 expression; therefore, the increase in ACE2 expression caused by these medications can be beneficial in reducing COVID-19 severity and progression [19]. Another study examined Wuhan cohorts and also found that ACEi and ARB use can be beneficial by inhibiting ACE2 receptors but can also upregulate ACE2 expression, which could potentially worsen COVID-19 infection. This study concluded that further research needed to be conducted regarding SARS-CoV-2 infection but that these medications have a beneficial effect on SARS-CoV [21]. 

As the contradictory studies discuss, it is possible that these antihypertensives can complicate COVID-19 infection and increase its severity because ACEis can increase bradykinin in tissue, which can lead to adverse effects, such as a dry cough, angioedema, increased vascular permeability, and vasodilation. This can potentially increase inflammation and worsen the prognosis in these COVID-19 patients on these medications [22]. 

However, these inflammatory effects from ACEi use can potentially be combated through the administration of ACEis via an alternative route. A clinical trial proposed that the efficacy of captopril nebulization in COVID-19 patients could be tested in a randomized study and hopes that patients with SARS-CoV-2 pneumonia could inhale this medication to increase ACE2 expression while maximizing lung action and reducing side effects [23,24]. The administration of these medications through this inhalation route could improve the overall prognosis of these patients when exposed to COVID-19. 

Other studies, particularly the Jia et al. study, brought to light some of the limitations of the aforementioned studies in this review that focused on the benefits of ACEis and ARBs. Similar to the findings of other studies discussed in the first portion of this review, the article by Jia et al. claimed that mortality may be decreased with ACEi and ARBs use in COVID-19 patients [25]. However, others in the scientific community have analyzed the findings of this study and found that the results were confounded by other variables. Scientists argue that the study's patient population sampled from November 2020 had greatly varying mortality rates compared to that of March 2020. Furthermore, they also claim there was no defined continuation and discontinuation period for the patients studied after discharge from the hospital. Mortality rates can vary at different points of the pandemic, so it is a challenge and a mistake to compare the effects of ACEis and ARBs at different times in the pandemic [26]. This study highlighted some potential limitations in the majority of the studies detailed in this literature review. Overall, further studies need to focus on comparing mortality rates of different months throughout the pandemic to rule out this confounder. 

## Conclusions

This review highlights the benefits of ACEi and ARB use in the treatment of symptoms associated with COVID-19 and long-term sequelae for those in recovery with cardiac comorbidities. Several articles were shown to demonstrate these findings and were explored in this literature review in addition to other studies that showed contradictory findings. This paper also discusses the limitations of these studies that showed the pros of utilizing ACEis and ARBs and discusses potential confounding variables. The variation in ACE2 gene expression in different ethnicities and the variation in COVID-19 mortality rates based on the time period chosen by the study are some examples of study limitations. Thus, future research needs to be conducted in these areas, particularly in ACE2 variants, to reduce the limitations in these studies. Because multiple studies have found some evidence of an epigenetic component, research in the next few years could provide a more detailed analysis. Likewise, further research is warranted to determine the long-term effectiveness of ACEis and ARBs since COVID-19, as it is a relatively novel area of research. This will better explain whether ACEi and ARB use serves benefits in reducing COVID-19 disease severity in the long term in patients with cardiac comorbidities. 
